# Defining fever: likelihood of infection diagnosis as a function of body temperature in the emergency department

**DOI:** 10.1186/cc14045

**Published:** 2014-12-03

**Authors:** E Small, CM Clements

**Affiliations:** 1 Department of Emergency Medicine, Mayo Clinic, Rochester, MN, USA

## Introduction

Fever predicts an infection cause of SIRS/sepsis more specifically than other commonly used hemodynamic criteria. Traditionally, a body temperature of 38.0°C has been used to define fever, but this cutoff is based on limited research. Furthermore, it has been proposed that fever responses are blunted in older adults, limiting the utility of fever in infection. This study determines the likelihood that a body temperature will predict diagnosis of infection in the emergency department (ED).

## Methods

This was a retrospective cohort analysis of adult patients (>18 years old) presenting to a large academic emergency department from September 2010 to December 2012. Patient age, emergency physician diagnosis, final disposition, and initial body temperature were examined for each patient. Likelihood of a diagnosis of infection was calculated for all temperature ranges. Sensitivity and specificity of fever thresholds for a diagnosis of infection were calculated and receiver operating characteristic (ROC) curves were generated. Confidence intervals were determined using Newcombe-Wilson hybrid scoring with continuity correction.

## Results

**Figure 1:**
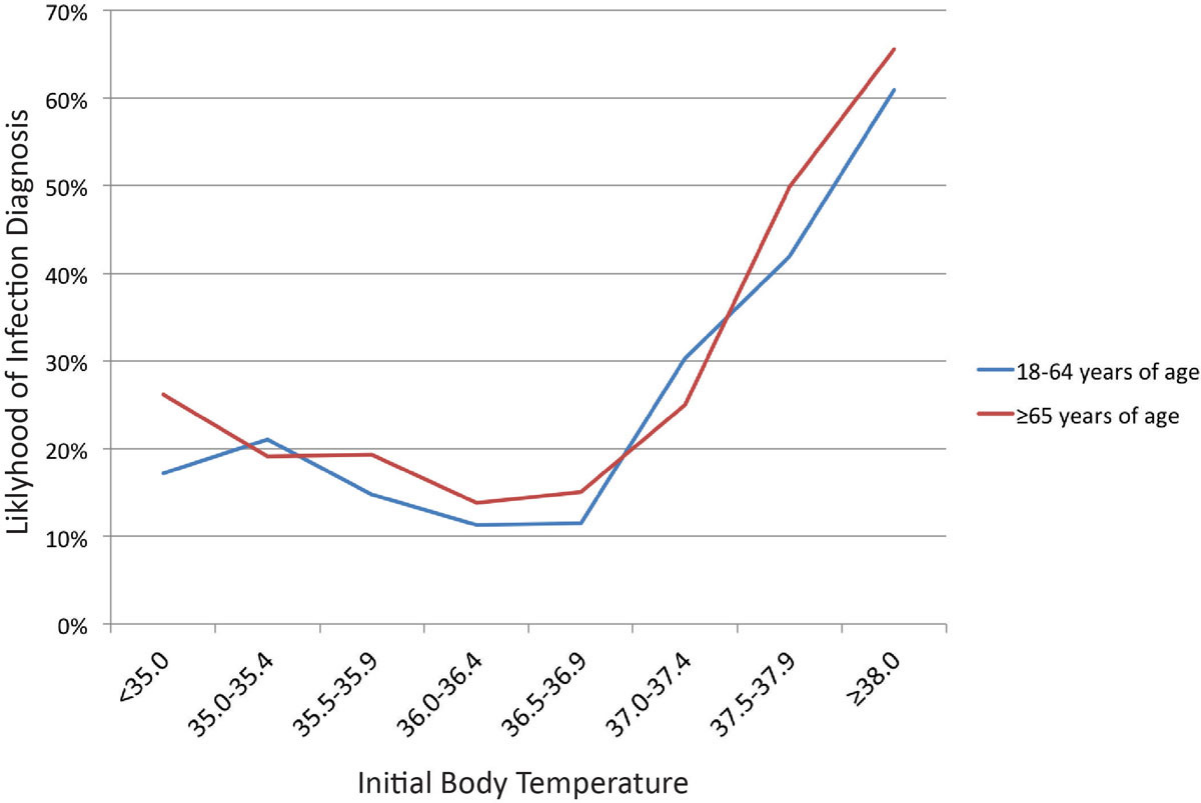
**Likelihood of infection diagnosis as a function of initial body temperature**.

We identified and analyzed records from 121,587 patients, including 37,933 persons >65 years old. Overall, 15.9% of patients received a diagnosis of infection in the ED with those >65 years old having a higher rate of infection at 18.3%. Likelihood of infection varied by temperature in a nonlinear relationship (see Figure 1). In adults of all ages, temperature >38.0°C had a specificity >99% for a diagnosis of infection in the ED although sensitivity was relatively poor. Specificity decreases only slightly with lower fever cutoffs (37.2 to 37.9°C) while sensitivity is increased to a greater degree (see Table 1).

**Table 1:** Sensitivity and specificity of body temperature for a diagnosis of infection.

	18 to 64 years of age		≥65 years of age	
	
	Sensitivity (95% CI)	Specificity (95% CI)	Sensitivity (95% CI)	Specificity (95% CI)
≥37.0	33.5% (32.6 to 34.3)	81.9% (81.6 to 82.2)	33.3% (32.2 to 34.4)	85.7% (85.3 to 86.1)
≥37.1	25.8% (25.0 to 26.6)	89.4% (89.1 to 89.6)	27.9% (26.8 to 29.0)	91.2% (90.8 to 91.5)
≥37.2	20.4% (19.7 to 21.2)	93.4% (93.2 to 93.5)	23.4% (22.4 to 24.4)	94.0% (93.7 to 94.3)
≥37.3	17.1% (16.4 to 17.8)	95.6% (95.5 to 95.8)	20.3% (19.3 to 21.2)	95.7% (95.5 to 95.9)
≥37.4	14.6% (14.0 to 15.3)	96.9% (96.8 to 97.0)	17.8% (16.9 to 18.7)	96.6% (96.4 to 96.8)
≥37.5	12.6% (12.0 to 13.2)	97.8% (97.7 to 97.9)	15.9% (15.0 to 16.7)	97.4% (97.2 to 97.6)
≥38.0	6.48% (6.06 to 6.93)	99.3% (99.2 to 99.3)	8.77% (8.12 to 9.47)	99.0% (98.9 to 99.1)

## Conclusion

The likelihood of a diagnosis of infection increases at the extremes of body temperature and is higher overall among older adults (≥65 years old). In our population, elevated temperature was at least as sensitive for a diagnosis of infection in older people as in younger adults. The use of body temperature for predicting diagnosis of infection has an overall low sensitivity but high specificity. Changing the current practical definition of fever from ≥38.0°C to ≥37.5°C significantly increases sensitivity of predicting infection without greatly impacting the specificity. Only 2.3% of all patients had a temperature between 37.5 and 37.9°C, and these patients were four times more likely to have a diagnosis of infection than those with temperature of 36.0 to 36.9°C.

